# Tumor budding as a risk factor of lymph node metastasis in submucosal invasive T1 colorectal carcinoma: a retrospective study

**DOI:** 10.1186/1471-2482-12-16

**Published:** 2012-08-06

**Authors:** Bong-Hyeon Kye, Ji-Han Jung, Hyung-Jin Kim, Se-Goo Kang, Hyeon-Min Cho, Jun-Gi Kim

**Affiliations:** 1 Department of Surgery, St. Vincent’s Hospital, College of Medicine, The Catholic University of Korea, 93-6, Ji-dong, Paldal-gu, Suwon-si, Gyeonggi-do, 442-723, South Korea; 2 Department of Pathology, St. Vincent’s Hospital, College of Medicine, The Catholic University of Korea, 93-6, Ji-dong, Paldal-gu, Suwon-si, Gyeonggi-do, 442-723, South Korea; 3 Department of Surgery, Seoul St. Mary’s Hopsital, College of Medicine, The Catholic University of Korea, 505 Banpo-dong, Seocho-gu, Seoul, Korea

**Keywords:** Lymph node metastasis, T1 colorectal cancer, Tumor budding

## Abstract

**Background:**

This study was designed to identify risk factors for lymph node metastasis of early stage colorectal cancer, which was confirmed to a carcinoma that invaded the submucosa after radical resection.

**Methods:**

In total, 55 patients revealing submucosal invasive colorectal carcinoma on pathology who underwent curative radical resection at the Department of Surgery, St. Vincent’s Hospital, The Catholic University of Korea from January 2007 to September 2010 were evaluated retrospectively. Tumor size, depth of submucosal invasion, histologic grade, lymphovascular invasion, tumor budding, and microacinar structure were reviewed by a single pathologist. Student *t*-test for continuous variables and Chi-square test for categorical variables were used for comparing the clinicopathological features between two groups (whether lymph node involvement existed or not). Continuous variables are expressed as the mean ± standard error while statistical significance is accepted at P < 0.05.

**Results:**

The mean age of 55 patients (34 males and 21 females) was 61.2 ± 9.6 years (range, 43–83). Histologically, eight (14.5%) patients had metastatic lymph node. In the univariate analysis, tumor budding (P = 0.047) was the only factor that was significantly associated with lymph node metastasis. Also, the tumor budding had a sensitivity of 83.3%, a specificity of 60.5%, and a negative predictive value of 0.958 for lymph node metastasis in submucosal invasive T1 colorectal cancer.

**Conclusions:**

The tumor budding seems to have a high sensitivity (83.3%), acceptable specificity (60.5%), and a high negative predictive value (0.958). A close examination of pathologic finding including tumor budding should be performed in order to manage early CRC properly.

## Background

An increase in colorectal cancer (CRC) screening and progress in techniques has resulted greater frequency of detection of T1 stage CRC. With recent advances in endoscopic techniques and improved endoscopes, T1 stage CRC and adenomatous polyps are often resected by endoscopes, regardless of their size or location [1-3]. Local treatments such as endoscopic resection and local excision are considered adequate management for early stage CRC without lymph node metastasis. A complete local treatment of intramucosal carcinoma is accepted as a curative treatment because there is little risk of lymph node metastasis. However according to the definitions used by most authors, colorectal adenomas with early invasive carcinoma contain a carcinoma that invades the submucosa, but not the muscularis propria. There is a risk of lymph node metastasis when cancer cells have invaded the submucosa. [3-6]. Therefore to avoid over-treatment or under-treatment, histopathological evaluation of locally excised specimens is important.

The previous reported incidence of lymph node metastasis in early stage CRC ranges from 0 to 15.4%, and the risk factors after endoscopic polypectomy include carcinoma at the surgical margin, lymphovascular invasion, poorly differentiated adenocarcinoma, and level of invasion[7,8]. Recently, some investigators suggested that a tumor budding is an another risk factor for lymph node metastasis of early stage CRC including occult metastasis [9,10].

This study was designed to identify risk factors for lymph node metastasis of early stage CRC, which was confirmed to a carcinoma that invade the submucosa after radical resection.

## Methods

A total of 55 patients revealing submucosal invasive colorectal carcinoma on pathology who underwent curative radical resection at the Department of Surgery, St. Vincent’s Hospital, The Catholic University of Korea from January 2007 to September 2010 were evaluated retrospectively. After obtaining the review board approval from our institute(VC11RISE0171), demographics and pathologic findings of 55 patients were reviewed retrospectively.

**Figure 1:**
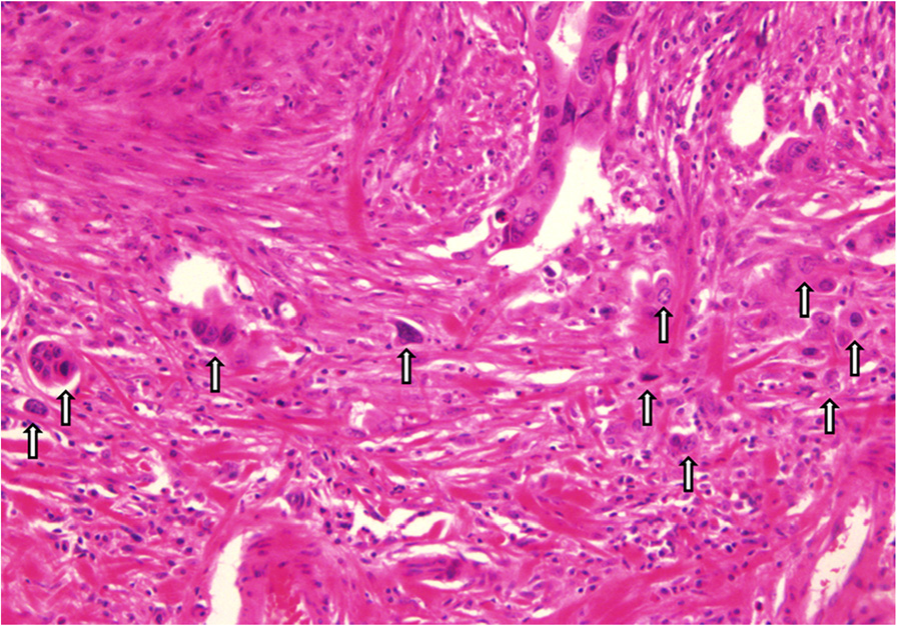
Representative hematoxyline-eosin (H & E) staining of tumor budding(arrow) - present case (x 200).

**Figure 2:**
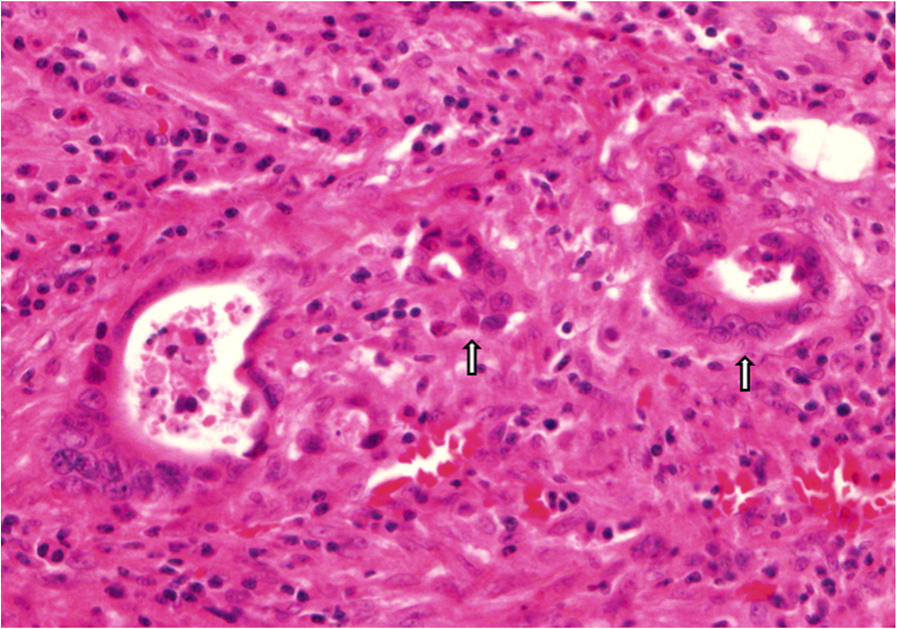
The tumor cells show microacinar structures (small round tubules) (arrow) (H & E x 400).

In this study, all tumors were macroscopically polypoid type which protruded above the surrounding surface at endoscopic finding. These tumors were classified into three different kinds: Ip, Isp, and Is. Ip is an abbreviation for pedunculated polyp which the base is narrow. Isp is an intermediate and broad-based forms. Lastly, Is is a sessile polyp which the base and the top of the lesion have the same diameter [11]. We defined the right sided colon as cecum, ascending, hepatic flexure, and proximal transverse colon. And the left sided colon was defined as mid to distal transverse, descending, sigmoid, and rectosigmoid colon. The circumferential ratio of the tumor represents the ratio of circumference of tumor to luminal circumference. All pathologic slides were re-examined in which tumor size, depth of submucosal invasion, histologic grade, lymphovascular invasion, tumor budding, and microacinar structure by a single pathologist. The depth of submucosal invasion was defined as the depth between muscularis mucosae and deep tumor margin measured in micrometers. Also, Kudo’s classification was used with the relative invasion depth of the submucosal layer and they are as follow: sm1, infiltration into the upper third of the submucosal layer; sm2, middle third; or sm3, lower third [4]. Histologic grade was classified in the order of well, moderately, poorly differentiated, and mucinous adenocarcinoma. Lymphovascular invasion is defined as the presence of tumor cells within small luminal structures lined by endothelial cells. An isolated cell or a small cluster of carcinoma cells in the invasive front is determined by looking at a budding focus, which when viewed at a 200-fold magnification, > 10 budding foci was considered as positive for tumor budding (Figure 1). Finally microacinar structure specifies small tubules that form cribriform structures within the medium or large glands, or small isolated round tubules within the stroma (Figure 2) [12]. 

Student *t*-test for continuous variables and Chi-square test for categorical variables were used for comparing the clinicopathological features between two groups whether lymph node involvement existed or not. Continuous variables are expressed as the mean ± standard error whereas statistical significance is accepted at P < 0.05. The applied statistical software was SPSS® 12.0 (SPSS Inc., Chicago, IL, USA).

## Results

There were a total of 55 patients including 34 males and 21 females. The mean age of the patients was 61.2 ± 9.6 years (range, 43–83). Histologically, eight patients (14.5%) had metastatic lymph node. The mean tumor size (the largest diameter) was 2.3 ± 1.4 cm (range, 0.7-8.0 cm). Table 1 shows the clinicopathological features in patients with early CRC with submucosal invasion. In the univariate analysis, the lymph node metastasis was significantly associated with the tumor budding (P = 0.047) while other factors were not statistically significant. Also, in the multivariate analysis, the tumor budding (P = 0.042, Hazard ratio 13.285, Confidence interval 1.094 – 161.297) was the only independent factor for lymph node metastasis in early CRC with submucosal invasion. The tumor budding had a sensitivity of 83.3% and a specificity of 60.5%. Also, although the tumor budding had a low positive predictive value (0.25), it had a negative predictive value of 0.958 for lymph node metastasis in submucosal invasive T1 colorectal cancer. Although there was not any statistically significant factor shown in the multivariate analysis, the depth of invasion by Kudo’s classification (P = 0.063) was a marginally meaningful predictive factor for lymph node metastasis in early CRC with submucosal invasion.

**Table 1:** Risk factor for lymph node metastasis in patients with submucosal invasive colorectal cancer

		**Lymph node involvement**		**P-value**	
		**No (n = 47)**	**Yes (n = 8)**	**Univariate**	**Multivariate**
Age	Mean ± S.D.	60.8 ± 9.7	64.0 ± 8.9	0.381	
Sex	male	29 (61.7 %)	5 (62.5 %)		
	female	18 (38.3 %)	3 (37.5 %)	0.966	
Tumor location	Rt. Sided colon^‡^	6 (12.8 %)	2 (25.0 %)		
	Lt. Sided colon^§^	22 (46.8 %)	4 (50.0 %)		
	Rectum	19 (40.4 %)	2 (25.0 %)	0.345	
Macroscopic shape of	Ip	23 (48.9 %)	3 (37.5 %)		
tumor	Isp	9 (19.1 %)	1 (12.5 %)		
	Is	12 (25.5 %)	3 (37.5 %)	0.485	
Tumor size	≤ 2.3 cm	20 (42.6 %)	5 (62.5 %)		
	> 2.3 cm	19 (40.4 %)	2 (25.0 %)	0.428	
Circumference ratio^†^	≤ 1/4	26 (55.3 %)	6 (75.0 %)		
	1/4 < or ≤ 2/4	17 (36.2 %)	2 (25.0 %)		
	2/4 < or ≤ 3/4	1 (2.1 %)	0		
	> 3/4	0	0	0.375	
Depth of invasion (μm)	< 1000	1 (2.1 %)	0		
	1000 ≤ or < 2000	6 (12.8 %)	2 (25.0 %)		
	2000 ≤ or < 3000	10 (21.3 %)	0		
	3000 ≤	23 (48.9 %)	4 (50.0 %)	0.435	0.282
Depth of invasion (by	Sm1	12 (25.5 %)	2 (25.0 %)		
Kudo’s classification)	Sm2	17 (36.2 %)	1 (12.5 %)		
	Sm3	10 (21.3 %)	3 (37.5 %)	0.364	0.063
Differentiation	well	21 (44.7 %)	1 (12.5 %)		
	moderately	21 (44.7 %)	6 (75.0 %)		
	poorly	0	0	0.112	0.856
Lymphovascular	Absent	37 (78.7 %)	4 (50.0 %)		
invasion	Present	5 (10.6 %)	2 (25.5 %)	0.206	0.232
Tumor budding	Absent	23 (48.9 %)	1 (12.5 %)		
	Present	15 (31.9 %)	5 (75.0 %)	0.047	0.042
Microacinar structure	Absent	29 (61.7 %)	3 (37.5 %)		
	Present	9 (19.1 %)	3 (37.5 %)	0.179	0.247

† = including cecum, ascending, hepatic flexure, and proximal transverse colon.

‡ = including mid to distal transverse, descending, sigmoid, and rectosigmoid colon.

§ = ratio of the circumference of tumor to luminal circumference.

In the present study, 22 of 55 patients underwent colonoscopic polypectomy before curative radical surgery. Table 2 shows the causes of radical surgery after colonoscopic polypectomy. An incomplete polypectomy (45.5%) was the most frequent reason for a curative radical surgery.

**Table 2:** Causes of radical resection after colonoscopic polypectomy

**Causes**	**N (=22)**
Involved or closed resection margin	10 (45.5 %)
Patient wanted (Sm1 or Sm2)	4 (18.2 %)
No identification of tumor depth, lymphovascular invasion, or resection margin	3 (13.7 %)
Lymphatic invasion (+)	2 (9.1 %)
No lifting of tumor at trial for polypectomy	1 (4.5 %)
Piecemeal resection	1 (4.5 %)
Sessile polyp c rectal invasive carcinoma	1 (4.5 %)

Table 3 shows the clinicopathological features of eight patients who had submucosal invasive CRC with lymph node metastasis. Data of malignant polyps in two patients who underwent colonoscopic polypectomy at a local clinic before radical surgery could not be found. The tumor budding was found in five (83.3%) of six patients whose pathologic data of malignant polyps could be found. The microacinar structure was found in three (50%) of six patients. There was one patient having a recurrence after curative radical surgery. She underwent laparoscopic low anterior resection for malignant rectal polyp located 7 cm from the anal verge. After the curative surgery, she was given an adjuvant chemotherapy using 5-FU and leucovorin (LF) regimen after curative surgery, but multiple unresectable liver metastases were found 3 months later. Although we gave FOLFOX chemotherapy, the liver metastases progressed and the patient passed away eleven months after radical surgery.

**Table 3:** Clinicopathological features of eight patients who had T1 colorectal cancer with lymph node metastases

**Sex**	**Age**	**Location**	**Shape**	**No of (+) LN**^**†**^	**Size (cm)**	**Grade**	**Depth**		**LVI**^**‡**^	**Budding**	**MA**^**§**^	**Recurrence site**	**DFS**^**∥**^**(months)**	**OS (months)**
							**μm**	**Kudo**						
F	60	S^††^	Isp	1	1.8	MD^†††^	5000	Sm3	-	+	+	No	54	54
M	64	Ra^‡‡^	Is	1	1.5	MD^†††^	1000	Sm1	-	+	+	No	49	49
M	63	S^††^	Is	1	1.5	MD^†††^	3500	Sm3	-	+	+	No	37	37
F	62	Rb^§§^	Is	6	3.2	MD^†††^	1500	Sm1	+	+	-	liver	3	11
M	75	A^∥∥^	Is	2	1.8	MD^†††^	3000	Sm3	-	-	-	No	15	15
F	78	A^∥∥^	Ip	4	6	WD^‡‡‡^	3000	Sm2	+	+	-	No	13	13
M	50	RS^¶¶^	Ip	2	3	MD^†††^	NR^§§§^	NR^§§§^	NR^§§§^	NR^§§§^	NR^§§§^	No	13	13
M	60	S^††^	Ip	1	1.6	MD^†††^	NR^§§§^	NR^§§§^	NR^§§§^	NR^§§§^	NR^§§§^	No	12	12

† = Lymph node, ‡ = Lymphovascular invasion, § = Microacinar structure, ∥ = Disease free survival,

¶ = Overall survival, †† = Sigmoid colon, ‡‡ = Rectum above peritoneal reflexion,

§§ = Rectum below peritoneal reflexion, ∥∥ = Ascending colon, ¶¶ = Rectosigmoid colon,

††† = Moderately differentiated, ‡‡‡ = Well differentiated, §§§ = Not reported.

## Discussion

The progress of endoscopic diagnosis and techniques has resulted higher frequency of detection of early stage CRC. With the advances of endoscopic instruments and techniques, the number of endoscopic resections for early-stage CRC has increased providing better quality of life for patients. In the treatment of early CRC which tumor cell forming in mucosal layer, it is better to perform a local treatment without adjuvant therapy because such cancer rarely metastasizes to the lymph node or distant organs [6]. With application of endoscopic treatments being widespread, an adverse outcome such as tumor recurrence was observed occasionally[13,14]. Therefore, this treatment is only adequate for early stage CRC without lymph node metastasis when the cancer cells have invaded submucosa [5-7]. In previous studies reported, the depth of submucosal invasion (Sm3), poor histologic grade, lymphovascular invasion, and tumor cell dissociation are labeled to be the risk factors for lymph node metastasis in submucosal invasive CRC.

Among these factors, the depth of submucosal invasion is an important predictor of lymph node metastasis [4-7,10,13-16]. There are two ways to measure the depth of submucosal invasion. First is to directly measure the vertical distance of submucosal invasion from muscularis mucosae. When the muscularis mucosae could not be identified due to carcinomatous invasion, the superficial aspect of the submucosal invasive carcinoma is used as a baseline. The other is by measuring a relative depth applying the Kudo classification [4]. Kitajima et al. [15] examined 865 submucosal invasive CRCs and analyzed the correlation between lymph node metastasis and depth of submucosal invasion. For pedunculated submucosal invasive CRC, the rate of lymph node metastasis was 0% in head invasion cases and stalk invasion cases with depth of invasion <3000 μm if lymphatic invasion was negative. For nonpedunculated submucosal invasive CRC, the rate of lymph node metastasis was also 0% if the depth of invasion was <1000 μm. Sung et al. [6] demonstrated that the minimal extent of submucosal invasion in tumors with lymph node metastasis was 1840 μm. Based on this finding, they suggested that when submucosal invasion of an endoscopically resected tumor was <1000-1500 μm, a complete cure can be achieved by endoscopic resection alone. Kudo et al. [4] proposed criteria for staging early CRCs in 1984 classifying degrees of submucosal invasion into three types based on the depth of invasion: sm1 (less than one-third of the submucosa is invaded), sm2 (intermediate), and sm3 (more than two-thirds of submucosa is invaded). For sm1, it is subclassified into sm1 into a, b, and c, according to the degree of horizontal extension of cancer in the submucosal layer. This staging of submucosal invasion is reflected to the prognosis of lesion. They reported that the lymph node metastasis was detected 3.7% in sm1c cancers, 10.9% in sm2 cancers, and 23.7% in sm3 cancers. In present study, eight patients had metastatic lymph node. However, data of malignant polyps in two patients who underwent colonoscopic polypectomy at a local clinic before radical surgery could not be found. There was no lymph node metastasis in submucosal invasive CRCs that the depth of invasion was <1000 μm. In terms of primary tumor with lymph node metastasis, four (67.7%) of six patients had a depth of invasion of ≥3000 μm while two patients had depth of 1000 μm and 1500 μm respectively. The lymph node metastasis was detected in 14.3% of sm1 cancers, 5.6% of sm2 cancers, and 23.1% of sm3 cancers. Comparing to other previous studies, the lymph node metastasis was more frequently detected in sm1 cancers. Based on this finding, although the depth of invasion is very important predictive factor for lymph node metastasis in submucosal invasive CRC, other factors such as microscopic grade, lymphovascular invasion, and tumor budding should be considered for complete cure by endoscopic resection alone. Ueno et al. [16] demonstrated that to extend the criteria for curative endoscopic resection, the combination of a quantitative factor and qualitative factors is useful. The quantitative factor is the depth of submucoal invasion and the qualitative factors include tumor grade, lymphovascular invasion, tumor budding, etc. Several studies have reported that the lymphovascular invasion is one of the histological risk factors for lymph node metastasis of submucosal invasive CRC[10,15-17]. In light of the clinical and biological importance of this feature in T1 tumors, American Joint Committee on Cancer recommended that the following modification of the T1 category (tumor invades the submucosa) to be used: T1a (no evidence of lymphatic or venous invasion) or T1b (lymphatic or venous invasion is present). However, it will remain problematic as long as diagnosis continues to differ occasionally among pathologists. Controversies over the detection of lymphovascular invasion arise mainly from the difficulty in visualizing the lymphatic vessel wall. It is difficult to detect lymphovascular invasion with conventional hematoxylin and eosin(HE) staining [18]. To overcome this limitation, Ishii et al.[19] recommended the use of immunostaining with some materials such as monoclonal antibody D2-40 demonstrating that such method would provide important information about the risk of lymph node metastasis and may prove useful in evaluating the necessity of an additional resection after local excision in T1 CRCs. In the present study, lymphovacular invasion was diagnosed in 7 (14.6%) of 48 cases that were examined only with HE staining. Lymph node metastasis could be found in 2 (28.6%) of these 7 cases. From our results, we conclude that a close evaluation for lymphovascular invasion with immunostaining may be helpful for identifying the risk factor for lymph node metastasis in submucosal invasive CRC. Tumor budding is also seen to be another important parameter in the evaluation of submucosal invasive CRC. This evaluation for tumor budding has merits in that evaluation is possible by ordinary HE staining [20]. Also, tumor budding may be a factor related to lymphovascular invasion. Morodomi et al. [21] have suggested that by making serial sections of specimen, budding is associated with lymphovascular invasion. Moreover, some reports suggest that the most important factor regarding the presence of budding is lymphovascular invasion.[22,23] High-grade tumor budding has been consistently linked to lymph node metastasis, distant metastasis, local recurrence and correlates with the distance of tumor invasion beyond the outer border of the muscularis propria. Tumor budding is proposed as a useful indicator for isolated tumor cells in lymph nodes in patients with early CRCs and could indicate curative surgery in patients with locally excised T1 tumors.[24] Also, microacinar structures (buds of tumor emanating from larger glands) and undifferentiated cells in stroma may be the antecedent stages of lymphatic invasion.[21] Goldstein et al.[12] suggested the association of extensive budding, microacinar structure and undifferentiated cells along the advancing edge with lymph node metastases. To sum up the findings, it is suggested that one of qualitative factors may not be detected independently, but any of those may be detected at progression of tumor. In other words, the detection of any qualitative factor is important in predicting lymph node metastasis. In this study, tumor budding (P = 0.042, Hazard ratio 13.285, Confidence interval 1.094 – 161.297) was the only independent factor for predicting lymph node metastasis. Also, although there was no lymph node metastasis in 15 patients with tumor budding (positive predictive value was 0.25) and not appropriated due to small sample size of this study, tumor budding seems to have a high sensitivity (83.3%), acceptable specificity (60.5%), and a high negative predictive value (0.958). We think that a high negative predictive value may be more important than a positive predictive value for clinician in order to decide whether an additional radical resection after local excision or colonocopic excision for submucosal invasive T1 colorectal cancer would be required or not. This finding may suggested that a close examination of histopathologic finding would provide important information about the risk of lymph node metastasis and may prove useful in evaluating the necessity of an additional resection after local excision in T1 CRC cases.

Our study does have some limitations. It was a retrospective study using the medical records of small number of patients, and the pathologic review of primary tumor could not be performed in all patients. Hence, our results for depth of invasion and lymphovascular invasion as a risk factor for lymph node metastasis were somewhat different compared with previous reported studies. Therefore, a large scaled prospective trial is necessary to verify the management strategy for submucosal invasive CRC.

## Conclusion

A close examination of pathologic finding including quantitative and qualitative factors should be performed for proper management of submucosal invasive CRC. Especially, the tumor budding seems to have a high sensitivity (83.3%), acceptable specificity (60.5%), and a high negative predictive value (0.958) in this study. Therefore, we think that the examination for tumor budding after colonoscopic or local excision for submucosal invasive CRC should be performed routinely. If any risk factor is detected, additional oncologic surgery would be necessary.

## Competing interests

The manuscript is an original work and has not been submitted or is under consideration for publication in another journal. The study complies with current ethical consideration. We also confirm that all the listed authors have participated actively in the study, and have seen and approved the submitted manuscript. The authors do not have any possible conflicts of interest.

## Author’s contributions

Study conception and design: Kye and Cho. Acquisition of data: Kye, Jung, HJ Kim, Kang, JG Kim, and Cho. Analysis and interpretation of data: Kye, Jung, HJ Kim, Kang, JG Kim, and Cho. Drafting of manuscript: Kye, HJ Kim, JG Kim, and Cho. Critical revision: Kye, Jung, HJ Kim, JG Kim, Kang, and Cho. Final approval : Jung, Kye, Kim, Kang, JG Kim, and Cho.

## Pre-publication history

The pre-publication history for this paper can be accessed here:

http://www.biomedcentral.com/1471-2482/12/16/prepub
